# sGC Activity and Regulation of Blood Flow in a Zebrafish Model System

**DOI:** 10.3389/fphys.2021.633171

**Published:** 2021-02-25

**Authors:** Krishan K. Vishnolia, Aleksandar Rakovic, Celine Hoene, Karim Tarhbalouti, Zouhair Aherrahrou, Jeanette Erdmann

**Affiliations:** ^1^Institute for Cardiogenetics, University of Lübeck, Lübeck, Germany; ^2^DZHK (German Research Centre for Cardiovascular Research), Partner Site Hamburg/Lübeck/Kiel, Lübeck, Germany; ^3^University of Lübeck, Lübeck, Germany; ^4^Institute of Neurogenetics, University of Lübeck, Lübeck, Germany

**Keywords:** *gucy1a1*, *GUCY1A3*, zebrafish, blood flow, soluble guanylate cyclase

## Abstract

Soluble guanylyl cyclase (sGC) protein is a heterodimer formed by two subunits encoded by *GUCY1A1* and *GUCY1B1* genes. The chromosomal locus 4q32.1 harbors both of these genes, which has been previously significantly associated with coronary artery disease, myocardial infarction, and high blood pressure. Blood pressure is influenced by both the environment and genetics and is complemented by several biological pathways. The underlying mechanisms associated with this locus and its genes still need to be investigated. In the current study, we aimed to establish the zebrafish as a model organism to investigate the mechanisms surrounding sGC activity and blood pressure. A zebrafish mutant *gucy1a1* line was generated using the CRISPR-Cas9 system by inducing a 4-bp deletion frameshift mutation. This mutation resulted in a reduction of *gucy1a1* expression in both heterozygote and homozygote zebrafish. Blood flow parameters (blood flow, arterial pulse, linear velocity, and vessel diameter) investigated in the *gucy1a1* mutants showed a significant increase in blood flow and linear velocity, which was augmented in the homozygotes. No significant differences were observed for the blood flow parameters measured from larvae with individual morpholino downregulation of *gucy1a1* and *gucy1b1*, but an increase in blood flow and linear velocity was observed after co-morpholino downregulation of both genes. In addition, the pharmacological sGC stimulator BAY41-2272 rescued the impaired cGMP production in the zebrafish *gucy1a1*^±^ mutant larvae. Downregulation of *cct7* gene did not show any significant difference on the blood flow parameters in both wild-type and *gucy1a1*^±^ background larvae. In summary, we successfully established a zebrafish platform for investigating sGC-associated pathways and underlying mechanisms in depth. This model system will have further applications, including for potential drug screening experiments.

## Background

High blood pressure (BP), or hypertension, is one of the most prominent heritable and preventable risk factors leading to premature death and the major cause for chronic heart failure, stroke, coronary artery disease (CAD), and kidney disease (Mills et al., 2016). According to the updated hypertension guideline, the estimated global financial burden of hypertension will reach approximately $200 billion by 2030 (DePalma et al., 2018). Currently, more than 1.3 billion people are estimated to have hypertension, which is expected to extend to 1.56 billion worldwide by 2025 (Kearney et al., 2005; Mills et al., 2016). Both the environment and genetics are contributing factors for BP (Evangelou et al., 2018). A recent genome-wide association study (GWAS) identified 535 novel loci associated with BP traits (systolic and diastolic blood pressure, and pulse pressure) in people of European ancestry drawn from the UK Biobank (UKB) and the International Consortium of BP-GWAS (ICBP) (Evangelou et al., 2018). The *GUCY1A1-GUCY1B1* locus (4q32.1) was associated with systolic and diastolic blood pressure by the ICBP (Ehret et al., 2011), and it was one of the five loci reported by the CARDIoGRAMplusC4D consortium showing significant association with BP along with CAD (Deloukas et al., 2013; Wobst et al., 2015).

Mechanisms catalyzing vascular retention of atherogenic lipoproteins and stimulated inflammation due to high blood pressure are well understood, along with impairment of nitric oxide (NO)-mediated vasodilation (Rippe et al., 2017). The latter plays a crucial role in regulating BP, and interference can lead to several clinical manifestations, such as stroke and chronically elevated BP, and in severe cases, myocardial infarction (Rippe et al., 2017). In hypertension, a reduction of NO-dependent vasodilation is partially attributed to decreased soluble guanylyl cyclase (*sGC*) protein levels that are recognized as a key NO receptor in the vascular wall of blood vessels (Rippe et al., 2017).

The sGC protein is a heterodimer comprised of α1 and β1 subunits, which are encoded by the genes *GUCY1A1* and *GUCY1B1* (previously known as *GUCY1A3* and *GUCY1B3*, respectively) (Erdmann et al., 2013). The sGC complex acts as key receptor for NO and promotes the generation of cyclic guanosine 3′, 5′-monophosphate (cGMP) (Erdmann et al., 2013; Wobst et al., 2015). cGMP regulates several physiological processes, such as proliferation and migration of vascular smooth muscle cells, neurotransmission, and inhibition of platelet aggregation (Erdmann et al., 2013; Wobst et al., 2015).

We previously conducted an extended family study to investigate the prevalence of premature CAD or myocardial infarction (MI) and identified a disease-causing variant in the *GUCY1A1* gene, which encodes for a subunit of sGC (Erdmann et al., 2013). The *GUCY1A1* frame shift mutation leads to a premature stop codon. Approximately 50% of carriers suffered with CAD and MI. In the same family, another rare mutation was identified in the *CCT7* gene encoding for a chaperonin stabilizing sGC (Erdmann et al., 2013). Individuals harboring both mutations were severely affected by CAD, suggesting a causal role of di-genic mutations. Further investigations revealed impaired sGC activity and significantly lower cGMP levels from platelets isolated from di-genic mutation carriers (Erdmann et al., 2013). In support of our findings, the α*1-* and β*1-*subunit protein levels were reduced *in vitro* after silencing *CCT7*, and *in vivo*, α*1-*subunit mutant mice showed an acceleration of occluding thrombus formation in the microcirculation after trauma (Erdmann et al., 2013).

In the past decade, synthetic sGC stimulators have entered the pharmaceutical market and proven beneficial in clinical trials for pulmonary arterial hypertension (Gheorghiade et al., 2015), chronic thromboembolic pulmonary hypertension (Ghofrani et al., 2013a), and heart failure, due to a reduced ejection fraction during heart contraction (Ghofrani et al., 2013b; Wobst et al., 2016). Previously, we identified a dose-dependent effect of the sGC stimulator BAY 41-2272 in the presence of NO on the inhibition of platelet aggregation. In cell culture setting (human embryonic kidney cells), different single nucleotide polymorphisms of the α1-subunit with significantly reduced protein levels were investigated by addition of BAY 41-2272 (Wobst et al., 2016). Treatment with BAY 41-2272 increased the production of cGMP without having an impact on protein levels (Wobst et al., 2016). Although there were several limitations to this study, these results provided a basis for impairment of sGC function in the mutated carriers of the *GUCY1A1* gene (Wobst et al., 2016).

A plethora of research has been conducted to investigate the different underlying mechanisms or characteristics of the sGC subunits, specifically the α*1-* and β*1-*subunits encoding for *GUCY1A1* and *GUCY1B1*, respectively, which have been previously associated by GWAS with hypertension, CAD, MI, and other diseases. However, there is still a demand for an animal model to perform high-throughput drug screening of potential drug candidates, which are currently only investigated *in vitro*.

The zebrafish (*Danio rerio*) is a well-established model organism used for disease modeling and drug screening because of several important characteristics: a high offspring output, genetic homology to the human genome, cheap and easy maintenance, optical transparency, and ease in genetic manipulation. Therefore, we determined that the zebrafish is a well-fitting candidate to study sGC and its associated phenotypes. So far, *GUCY-1A1* and *GUCY*-*1B1* have been mostly studied using live cell lines, mice for disease phenotypic studies, or in clinical trials on humans. In the current study, we aim to establish the zebrafish as a model for future sGC subunits studies by investigating different phenotypes associated with the two subunits.

## Materials and Methods

### Zebrafish Maintenance

All zebrafish experiments were ethically approved and conducted in accordance with the guidelines of the Animal Studies Committee in Schleswig-Holstein, Germany. AB-wild-type zebrafish were purchased from European Zebrafish Resource Center (EZRC, Germany) and maintained at the Fraunhofer Institute of Marine Biotechnology (EMB, Lübeck, Germany) as described in The Zebrafish book (Westerfield et al., 1997; Westerfield, 2000).

### Generation of Zebrafish *gucy1a1* Knockout Line

**FIGURE 1:**
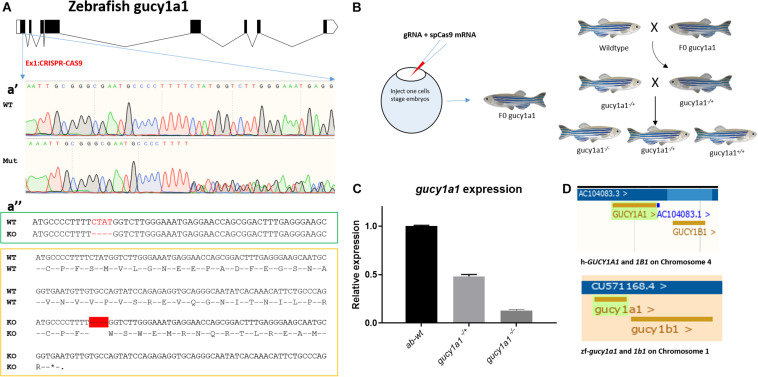
**(A)** Schematic of zebrafish *gucy1a1* gene presenting all the exons in box forms and introns in the form of connecting lines, **a’** showing sequencing result comparison of zebrafish wild-type allele (WT) vs. mutant (Mut) at gRNA target site on *gucy1a1* gene exon 1, **a”** (in the green box) highlights 4 base pair deletion (highlighted as red dashes) at the mRNA level in the knockout zebrafish compared to the wild types, and **a”** (in the yellow box) shows the deletion of 4 base pair leading to premature stop codon at the amino acid level after 16 amino acids. **(B)** Schematic of gRNA + spCas9 mRNA in one-cell stage zebrafish embryos leading to identification of F0 founder fish, which were out crossed with AB wild-types following which heterozygotes were identified and in crossed. **(C)** mRNA relative expression of *gucy1a1* gene was observed significantly reduced in the *gucy1a1^+/–^* (±50%) and *gucy1a1^– /–^* (±90%), error bars represent ±SD. **(D)** Schematic represents the conserved genomic positioning of both *gucy1a1* and *gucy1b1* genes in human and zebrafish genome, screen shots taken from Ensemble genome browser.

To generate *gucy1a1* knockout line, one-cell zebrafish embryos were co-injected with 200 ng of *in vitro* synthesized guide RNA (gRNA) targeting the GGGCGAATGCCCCTTTTCTA sequence in the first exon of the *gucy1a1* gene (Figure 1A) and 400 ng of *in vitro* synthesized Cas9 mRNA. After 2 months, fish were fin-clipped and genomic DNA was extracted. The targeted genomic locus was amplified using the following primers: Forward GTTTGGAGAGGACCATTACGC and Reverse TTTACCTCTGGACACACCAGC, designed to anneal upstream and downstream from the targeted region and sequenced using Sanger sequencing (Figure 1Aa’). Only fish carrying indels (classified as potential founders) were kept to reach sexual maturity. Each potential founder was crossed with the AB-wild-type zebrafish. Four days post-fertilization, 20 embryos from each potential founder were lysed individually with 25 μl of the proteinase K buffer (1 μg/μl proteinase K in 1xPCR buffer) and incubated for 50 min at 55°C, followed by inactivation at 95°C for 10 min. Extracted DNA was screened for the presence of indel mutations by PCR using the above-mentioned primers, followed by Sanger sequencing.

Finally, the offspring of the confirmed founder were maintained for 2 months and fin-clipped to identify animals carrying identical frameshift mutations in the *gucy1a1* gene. As presented in the schematic in Figure 1B, *gucy1a1*^±^ were crossed to obtain *gucy1a1*^+/+^, *gucy1a1*^±^, and *gucy1a1*^–/–^ for all the further experiments.

### Morpholino Downregulation

One-cell zebrafish embryos of both AB-wild-type and *gucy1a1* mutants were injected with approximately 5 ng of transcription splice modification morpholinos of *gucy1a1*-e2i2 (AAAAGAACATCTTACCAACTCGTGT), *gucy1b1*-e2i2 (TATTTTTGAACTCACTTAATGTCCT), and *cct7*-e2i2 (ATACAGAATTGCTGACTAACCTCGG) along with a gene-specific control morpholino as previously described (Westerfield, 2000). Morpholinos were injected individually or in conjunction with other morpholinos as per the requirements of the experiment.

Following blood flow measurements from individual larvae, the morpholino downregulation efficiency at the mRNA level was assessed using qPCR analysis. Total RNA was extracted using RNeasy kit (Qiagen, Germany) following the manufacturer’s protocol. *EF1*α and β*-actin* were used as internal housekeeping genes, and the custom-designed primer sequences are described in Table 1. qPCR data were analyzed using the comparative threshold cycle method (2^–Δ^ ^Δ^ ^Ct^) to compare expression levels in samples and controls, where control samples were set to a value of 1.

**TABLE 1:** Sequences for all the primers (both forward and reverse) along with their respective purpose in the present study.

Gene name	Sequence	Purpose	PCR product size	Accession number
*gucy1a1*-F	GTTTGGAGAGGACCATTACGC	Sequencing	265	NM_001309529.1
*gucy1a1*-R	TTTACCTCTGGACACACCAGC	Sequencing		
*gucy1a1*-F	GGACAAAGATCCAGGGCTCC	MO qRT-PCR	156	NM_001309529.1
*gucy1a1*-R	AGAGCGCCACTGTCTTTACC	MO qRT-PCR		
*gucy1b1*-F	AGTGTTGGGCTCCAATGTCC	MO qRT-PCR	212	NM_001251945.2
*gucy1b1*-R	CGTGAATCTGCTGTGCAACC	MO qRT-PCR		
*cct7*-F	GGTGGGGCAATTGAGATGGA	MO qRT-PCR	198	NM_173248.1
*cct7*-R	CCACATACCACCCTGTGCAT	MO qRT-PCR		
*EF1*α*-*F	CTGGAGGCCAGCTCAAACAT	HKG	87	NM_131263.1
*EF1*α*-*R	ATCAAGAAGAGTAGTACCGCTAGCATTAC	HKG		
β*-actin*-F	CGAGCTGTCTTCCCATCCA	HKG	86	NM_181601.5
β*-actin*-R	TCACCAACGTAGCTGTCTTTCTG	HKG		

### Hypertension Parameters

Blood flow parameters, i.e., arterial pulse, blood flow, linear velocity, and vessel diameter (surrogate parameters for blood pressure), were measured following the same protocol as in previous publication (Parker et al., 2014; Vishnolia et al., 2020). Briefly, 72 h post fertilization (hpf) zebrafish larvae were mounted on glass coverslips using 0.1% low melting agarose gel maintained at 17°C. Twenty-minute video files were recorded at 120 frames per second (fps) at room temperature using an inverted microscope (Zeiss stereo, Discovery V20, Germany), mounted with high-speed video cameras (GRAS-03K2M-C, Point Grey Research Inc., Richmond, Canada) focused on dorsal aorta, caudal to swim bladder.

Video files from the positive genotyped (*n* = 12) and control zebrafish (*n* = 10) larvae were analyzed using MicroZebraLab software (Version 3.6, ViewPoint, Lyon, France). After software calibration using a video of a hemocytometer grid, the software detects pixel density along with vessel diameter to compute the flow rate (nl/sec) per frame. An area of interest on the aortic blood vessel was selected carefully, avoiding nearby capillaries as they might interfere in determining the blood flow in the main vessel. Vessel diameter was determined every 20 s by the software following manual selection of vessel and confirmation of two vessel edges. Blood flow was computed by the software by determining the movement of erythrocytes within the region of interest. To let the zebrafish larvae acclimatize to the conditions, analysis from the first 3 min of every video file was excluded from the results as demonstrated in previous literature (Parker et al., 2014) and reported in our publication previously (Vishnolia et al., 2020).

### Bay41-2272 Stimulation and cGMP Measurements

Cyclic guanosine 3′, 5′-monophosphate levels were measured from the homogenized lysate of zebrafish larvae using the cGMP ELISA kit (Cyclic GMP ELISA kit-581021, Cayman chemicals) following the manufacturer’s protocol. Zebrafish *gucy1a1*^±^ and wild-type larvae (*n* = 30 larvae per group) at 4 days post-fertilization were synergistically treated for 1 h with H_2_O, 1, 5, and 10 μM of BAY 41-2272 (B8810, Sigma-Aldrich, Germany) sGC stimulator. All larvae were snap frozen in liquid nitrogen (in groups of 10) before being homogenized in the ELISA buffer using a hand-held motorized homogenizer. Tissue sample lysates along the standards were carefully acetylated following the manufacturer’s protocol to enhance sensitivity and avoid interference from other contaminants. Following the instructions of the kit, the ELISA plate was prepared, developed, read at a 405 nm absorbance wavelength (BioTek instruments, United States), and cGMP values were calculated.

### Statistical Analysis

Statistical analysis was performed using the GraphPad Prism 6 program. Data were tested for normal distribution following which non-parametric Student’s *t*-test was used to analyze the data (represented as mean ± SD) from experiments with only two groups. A two-way ANOVA with multiple comparisons and without corrections was used when experiments contained three or more groups for analysis. The significance alpha *p*-value was adjusted by dividing it with the total number of tests performed.

## Results

### Conservation of Human *GUCY* Subunits in Zebrafish

Both the *GUCY-1A1* and *GUCY*-*1B1* subunits of sGC are located at locus 4q32.1 on chromosome 1. Data mining on the ensemble genome browser database revealed identical genomic positioning of both *gucy1a1* and *gucy1b1* genes between human and zebrafish genomes, respectively, as shown in Figure 1D. Both the genes were also identified to be highly conserved in human and zebrafish such as *gucy1a1* gene having 95% coverage and 58% identical protein (Browser, 2020a), whereas *gucy1b1* having 98% coverage and 88% identical protein (Browse, 2020b) (as of Ensemble release 102-November 2020).

### Generation of Zebrafish *gucy1a1* Mutant Line

We generated a *gucy1a1* mutant using AB-wild-type background zebrafish by inducing a 4-base-pair frame-shift deletion mutation in the exon 1 of the *gucy1a1* gene using the clustered regularly interspaced short palindromic repeat (CRISPR)-Cas9 technology (Figures 1A,B). Sequencing chromatograph shown in Figure 1Aa’ represents a mixture of different sequences after “TTTT,” i.e., wild-type, mutant sequences.

The frame-shift deletion of 4 base pairs led to the formation of a stop codon after 16 amino acids as shown in Figure 1A. mRNA expression analysis showed a 50% ± 10% and 90% ± 10% reduction in *gucy1a1* heterozygous and homozygous mutation carriers, respectively, when compared with the wild-type siblings (Figure 1C). We observed no gross morphological differences between wild-type and *gucy1a1* mutant fish at either larval or adult stage. We did not had success in generating the *gucy1b1* and *cct7* zebrafish mutant line using same approach, and therefore, we employed the morpholino downregulation approach for *gucy1b1* and *cct7* gene in the current study.

### Increased Blood Flow Measurements in *gucy1a1* Mutants

**FIGURE 2:**
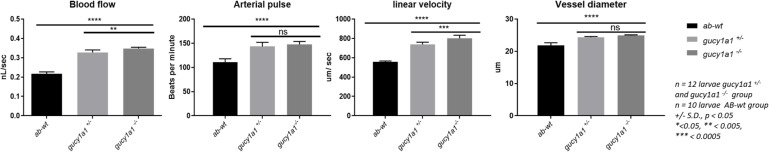
Blood flow parameters measured from AB-wild types, heterozygote and homozygote for *gucy1a1* gene in zebrafish larvae. Blood flow (nL/sec), arterial pulse (beats per minute), linear velocity (μm/sec), and vessel diameter (μm) were measured from *gucy1a1^+/–^* (*n* = 12), *gucy1a1^– /–^* (*n* = 12), and AB-wild-type (*n* = 10) larvae per group. Data are presented as mean ± standard deviation, significance was set at *p* < 0.05, and stars denote the significant differences.

We observed a significant increase in blood flow, arterial pulse, linear velocity, and vessel diameter, measured after 72 hpf, in both *gucy1a1*^±^ (*n* = 12) and *gucy1a1*^–/–^ (*n* = 12) mutants compared with AB-wild-type controls (*n* = 10) (Figure 2). Both blood flow and linear velocity parameters were significantly higher in the homozygotes than in the heterozygotes. However, no significant differences were found in the arterial pulse or vessel diameter parameters between heterozygotes and homozygotes, as shown in Figure 2.

### *gucy1a1, gucy1b1*, and *cct7* Morpholino Downregulation

**FIGURE 3:**
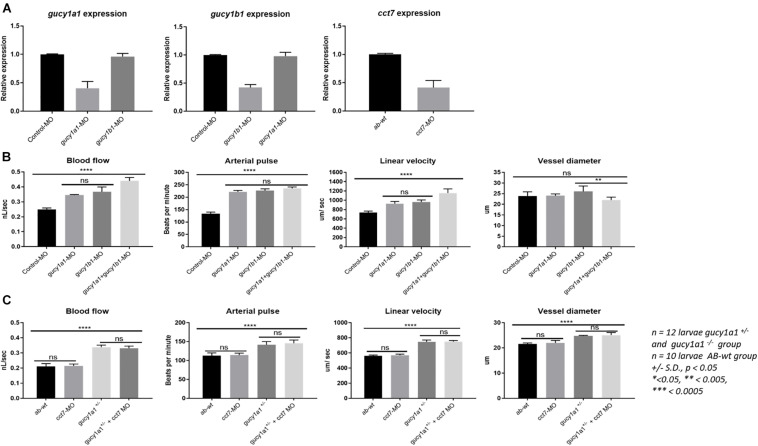
**(A)** Graphs represent mRNA downregulation of *gucy1a1, gucy1b1*, and *cct7* genes using morpholino (MO) in zebrafish. No cross reactivity of morpholinos was observed between the two subunits of *gucy* gene in the mRNA expression. **(B)** Graphs represent comparison of blood flow parameters measured from *gucy1a1* (*n* = 12), *gucy1b1* (*n* = 12), *gucy1a1+gucy1b1* (*n* = 12), and control-MO (*n* = 10). **(C)** Graphs presenting comparison of blood flow parameters measured from AB-wild-type (*n* = 10), *cct7*-MO (*n* = 12), *gucy1a1^+/–^* (*n* = 12), and *gucy1a1^+/–^* + *cct7*-MO (*n* = 12). Data are presented as mean ± standard deviation, significance was set at *p* < 0.05, and stars denote the significant differences.

qPCR analysis showed approximately 60–65% knockdown of the *gucy1a1*-, *gucy1b1-*, and *cct7-* injected morpholinos, relative to control. No cross-reactivity of the morpholinos was observed. In particular, no downregulation was observed of the *gucy1b1* subunit in the *gucy1a1*-injected morpholino larvae or *gucy1a1* subunit in the *gucy1b1*-injected morpholino (Figure 3A).

### *gucy1a1* and *gucy1b1-*Morpholino Combined Effect

Although there was significant increase in the blood flow parameters in morpholino-injected *gucy1a1* and *gucy1b1* subunits compared to control morpholino-injected larvae, no significant differences were observed in the blood flow parameters among the two morpholino downregulated *gucy1a1* and *gucy1b1* (*n* = 12 larvae per group) subunits injected individually as shown in Figure 3B. Interestingly, when morpholinos for both subunits were co-injected, we observed a significant increase in the blood flow and linear velocity, but a decrease in the larval vessel diameter compared to individually morpholino downregulated subunit (Figure 3B). Decrease in vessel diameter was not significant between the control morpholino-injected and *gucy1a1* and *gucy1b1* morpholino co-injected larvae. Arterial pulse did not change in larvae co-injected with both morpholinos compared to individual injected morpholino for both subunits; however, consistent significant increase was observed in morpholino co-injected larvae compared to control morpholino-injected larvae.

### Effect of *cct7* Downregulation in *gucy1a1*^±^ Mutants on Blood Flow Parameter

Injection of *cct7* morpholinos into AB-wild-type zebrafish embryos presented no differences in the blood flow parameters (Figure 3C). Interestingly contrary to the familial study previously published by our group (Erdmann et al., 2013), *gucy1a1*^±^ zebrafish embryos injected with the *cct7* morpholino induced no change in blood flow parameters, in contrast to the *gucy1a1*^±^ control (Figure 3C).

### sGC Stimulator Dependent Increment in cGMP Levels

**FIGURE 4:**
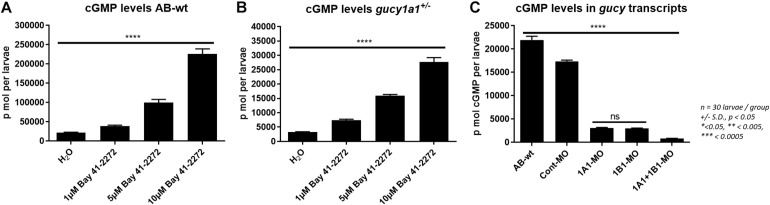
**(A)** Graph presents cGMP levels (p mol per larvae) measured from AB-wild-type larvae at 4 dpf treated with H_2_O, 1, 5, and 10 μM BAY41-2272 concentration. **(B)** cGMP levels measured and compared from *gucy1a1^+/–^* treated with H_2_O, 1, 5, and 10 μM BAY41-2272 concentration. **(C)** cGMP levels measured and compared between AB-wild types, morpholino injected controls, *gucy1a1-*MO, *gucy1b1*-MO, and *gucy1a1+gucy1b1*-MO. Data are presented as mean ± standard deviation from *n* = 30 larvae per group, significance was set at *p* < 0.05, and stars denote the significant differences.

Cyclic guanosine 3’, 5’-monophosphate levels were measured from AB-wild-type zebrafish larvae treated with either H_2_O or 1, 5, or 10 μM concentrations of BAY 41-2272 stimulator. The cGMP levels of the larvae increased significantly in a dose-dependent manner (Figure 4A). The cGMP level was also observed to increase in a dose-dependent manner in *gucy1a1*^±^ zebrafish mutants (Figure 4B). However, the cGMP levels were approximately 10-fold higher in the wild-type than in mutants.

Further, we found no significant difference in the cGMP levels between the two knockdowns of the *gucy1a1* and *gucy1b1* morpholinos (Figure 4C). We noticed a significantly lower cGMP level when morpholinos for both the subunits were co-injected in the zebrafish larvae (Figure 4C).

## Discussion

In the current study, we aimed to establish zebrafish as a model system for studying the underlying mechanisms for sGC and its subunits. The zebrafish *gucy1a1* mutant line was created to explicitly study the phenotypes associated with this gene, with specific interest in the blood flow parameters. Significantly increased blood flow, arterial pulse, linear velocity, and vessel dilation were observed in the mutant larvae compared with the wild type. The increased blood flow parameters in the *gucy1a1* mutants are consistent with predicted associations from GWAS (Ehret et al., 2011; Evangelou et al., 2018) and previously published data (Wallace et al., 2016).

It is further warranted to investigate the effect of the gene encoding β-subunit, i.e., *gucy1b1*, in the sGC heterodimer. As we were unsuccessful in creating the zebrafish mutant line for *gucy1b1*, we employed the morpholino approach. Successful significant downregulation (50–60%), without any cross-reactivity among the two subunit morpholinos, was observed, leading to significantly increased blood flow parameters when injected individually. By comparison, larvae co-injected with both the *gucy1a1* and *gucy1b1* morpholinos significantly increased blood flow and linear velocity; however, no such significant difference was observed for arterial pulse. Surprisingly, we did not observe any significant difference in vessel diameter between larvae injected individually with morpholinos compared with controls. By comparison, larvae co-injected with both subunit morpholinos showed a reduced vessel diameter compared with those injected with *gucy1b1* morpholino.

Impaired physiological function of the vascular wall is a very common characteristic in the diseased state, leading to either vasodilation or vasoconstriction due to anaphylactic shock or hypertension (Lerman and Burnett, 1992). Endothelial dysfunction is a crucial factor in CADs such as hypertension, MI, and atherosclerosis, which predominantly lead to impaired NO-sGC-cGMP signaling (John and Schmieder, 2003). Several factors could be accountable for the effect on the NO-sGC-cGMP pathway, such as (1) reduced NOS activity due to reduced NO availability, (2) increased PDE activity that could fast-track cGMP degradation (Wilcox, 2002; John and Schmieder, 2003; Stasch et al., 2006), (3) oxidative stress leading to reduced free radicals to scavenge NO (Wilcox, 2002), or (4) downregulation of sGC or cGMP targets (Boerrigter and Burnett Jr., 2007). Therefore, we aimed to investigate if the well-established pharmacological sGC modulator (BAY 41-2272) could rescue the impaired cGMP formation in our *gucy1a1*^±^ zebrafish system. Indeed, treatment of *gucy1a1*^±^ mutants with BAY 41-2272 presented a significant increase in the cGMP levels respective to the treatment concentration. This result is supported by previous *in vitro* studies (Boerrigter and Burnett Jr., 2007; Wobst et al., 2016) and provides evidence that the zebrafish is an ideal *in vivo* model system to examine the mechanisms associated with sGC and its subunits.

Mutations in the *GUCY1A1* gene leads to truncated or non- functional proteins, which has been linked with higher genetic risk for CAD/MI. Individuals carrying a *cct7* mutation, a gene that encodes the chaperone protein, leads to premature CAD/MI in a previously reported extended family study (Erdmann et al., 2013). In our study, injection of the *gucy1a1*^±^ zebrafish larvae with the *cct7* morpholino did not show any significant differences in the BP parameters, compared with the *gucy1a1*^±^ larvae. Nevertheless, the *cct7* morpholino-injected zebrafish larvae did not show any change in the blood flow parameters compared with controls. We believe that this needs to be further investigated to understand the effect of *cct7* downregulation; however, a complete *cct7* knockout mutant line should be used. To investigate and understand the mechanisms relating to atherosclerosis, the zebrafish mutants (*gucy1a1, gucy1a1, gucy1a1* + *cct7, gucy1a1* + *gucy1b1, gucy1b1* + *cct7*, and *gucy1a1* + *gucy1b1* + *cct7*) could be treated with high cholesterol diets to measure the effects on cholesterol levels, lipid deposits, and downstream effects of the PDEs.

In summary, we have shown that the zebrafish model system can successfully recapitulate the sGC-associated phenotypes including blood flow parameter alterations. The *gucy1a1*^–/+^ zebrafish mutants demonstrated impaired cGMP formation and a combined effect of the α1- and β1-subunits on the blood flow parameters when downregulated by morpholino injection. Nevertheless, further investigation is warranted to produce a complete knockout mutant line for the *cct7* and *gucy1b1* genes in the zebrafish. Overall, this model system could serve as an ideal platform for future studies to investigate the underlying mechanisms of sGC activity and have further applications for potential drug screening experiments.

## Data Availability Statement

The raw data supporting the conclusions of this article will be made available by the authors, without undue reservation.

## Ethics Statement

Ethical approval of the animal study was waived in accordance with the recommendations of guidelines, EU Directive 2010/63/EU, set by the European Commission, and according to this legislation, embryos and larvae up to 5 days old are excepted. The legislation criterion is independently feeding larval forms (Directive 2010/63/EU), in other words, when the larvae are able to move through the water column independently, when their digestive tract is functional, and when they are beginning to hunt for prey which has also been simplified and published in PMID: 21726626. For generation of zebrafish *gucy1a1* mutant line, approval was granted by the Animal Ethical Committee of Schleswig-Holstein, Germany, and the reference number for that is V 242-7224.122-39 (77-6/14).

## Author Contributions

KV conceived, designed, performed, and collected the data, and wrote the manuscript. AR contributed to generating the *gucy1a1* knockout line. CH and KT contributed to critical analysis of data and manuscript revisions. ZA and JE supervised the project, critically analyzed the data, reviewed the manuscript, and helped in designing the initial concept of the project. All authors contributed to the article and approved the submitted version.

## Conflict of Interest

The authors declare that the research was conducted in the absence of any commercial or financial relationships that could be construed as a potential conflict of interest.
